# Berries, greens, and medicinal herbs—mapping and assessing wild plants as an ecosystem service in Transylvania (Romania)

**DOI:** 10.1186/s13002-020-0360-x

**Published:** 2020-03-04

**Authors:** Ágnes Vári, Ildikó Arany, Ágnes Kalóczkai, Katalin Kelemen, Judith Papp, Bálint Czúcz

**Affiliations:** 1 MTA Centre for Ecological Research, Institute of Ecology and Botany, Vácrátót, Hungary; 2 MTA Centre for Ecological Research, GINOP Sustainable Ecosystems Group, Tihany, Hungary; 3 Milvus Group Association, Crinului Str. 22, 540343 Târgu Mureş, Romania

**Keywords:** Wild edible plants, Wild food plants, Non-timber forest products, Mapping, Spreadsheet approach, Human well-being, Motivations

## Abstract

**Background:**

Wild edible plants as well as medicinal herbs are still widely used natural resources in Eastern Europe that are frequently accessed by the local population. Ethnobotanical studies rarely give insight to the specific ecosystems in which wild food and medicinal plants grow in a spatially explicit way. The present work assesses the potential of different ecosystems to provide wild plants for food and medicinal use based on 37 selected plant species, gives an estimate on the actual use of wild plants, and allows insights into the motivation of local people to collect wild plants.

**Methods:**

A number of interdisciplinary methods were used: participatory stakeholder workshops with experts scoring the provisioning capacity of ecosystem types, GIS for representing results (capacity maps), basic data statistics for actual use assessment, and interviews for analysing motivations.

**Results:**

Capacity to provide wild edible plants was assessed highest in broad-leaved forests and wetlands, while for medicinal herbs, orchards were rated best. We could find a multitude of motivations for gathering that could be grouped along four main lines corresponding to major dimensions of well-being (health, habit/tradition, nutrition/income, pleasure/emotional), with health reasons dominating very clearly the range (59% of answers), which can be interpreted as a combination of modern “green” values with a traditional lifestyle. We detected some distinct patterns of motivations between the different social groups analysed with more fundamental needs associated with lower level socio-ecological background.

**Conclusion:**

This case study provides an example on the importance of wild plants for locals from several points of view. We emphasize the relevance of these local stakeholder views to be included in decision-making and ecosystem management, which can be achieved by the presented workflow for mapping and assessment of ecosystem services which is also compatible with EU-suggested Mapping and Assessment of Ecosystems and their Services (MAES).

## Background

Plants collected from the wild have been used since ancient times [1, 2] and constitute still a vivid link to nature in some areas, even in highly human-impacted regions, like Europe [2–6]. While originally a basic way to fulfil several key human needs (food, medicine), nowadays, a diversity of reasons can be named for people still relating to this activity, with one major line being strong cultural bonds [4, 7–9]. Wild plants can be used as food, medicine, material, fuel, for ornamental, or even for “magical” purposes [10]*.* Wild plants can thus be rightfully seen as an ecosystem service, or even as a collection of ecosystem services, as “wild plants” is, in fact, an umbrella term covering many different concrete goods, each of which can be seen as a different service. Essentially, these goods (species and species parts collected) do not have any trivial “common metric”, meaning that a certain amount (e.g. kg mass) of one good (e.g. truffles) is not equal to the same amount of the other (e.g. stinging nettles).

Ecosystem service assessment is a tool which integrates diverse information for management purposes. It can incorporate a wide variety of data and be at different levels of complexity, depending on data (and model) availability [11]. The ES framework aims to offer a common basis for comparing very different types of services with each other and synthesize their aggregated importance. Local knowledge is also often included in ES assessments with a participatory approach (e.g. [12–14]). Local communities collecting and consuming wild plants have a century-old traditional ecological knowledge on these services and the ecosystems providing them [15, 16]. Bringing the ES aspects to the topic of wild plants promises a different type of insights, potentially more valuable for underpinning management options than a merely top-down approach.

Wild plants are not only neglected in statistics on food production, but they are also underrepresented in recent ES assessments [1, 3, 17–19]. This is probably partly due to the fact that many prominent ES classifications fail to distinguish “wild” resources from cultivated crops as distinct classes of provisioning services (e.g. [20, 21]). Nevertheless, wild food as an ecosystem service is not only a provisioning service, but it can also be perceived as a cultural service through the recreational components of the activity (e.g. berry picking) or even contributing to the cultural identity of the local communities (e.g. [3, 5, 22]).

The collection of wild plants is mainly covered by ethnobotanical surveys, giving plant species lists and describing possible uses of the plants in detail [10, 23–26]. A significant share of the literature focuses on Asia or the Middle-East (e.g. [27–31]). There are also several studies from Europe, especially for Scandinavia [5, 32–34], or for greens and fruit in general in Spain [4, 8, 35, 36]. The Hungarian-speaking minorities in the Carpathian Basin, and in Romania specifically, are a relatively well-studied group from an ethnographic perspective, including ethnobotanical studies ([37] and literature cited therein). Dénes et al. [38] give a thorough overview on wild food plants, while a number of works tackle certain aspects, e.g. focus on ethnomedicine [39].

Only few studies attempt to locate plants related to certain habitats and/or categorize the habitats themselves (but see [22, 40] for urban environments, or [41, 42]). Mapping, as a spatially explicit representation of the obtained results, is rarely undertaken. There are some studies on Scandinavian forests and their non-timber forest products that elaborate yield models with a diverse set of habitat characteristics [34, 43].

Studying the motivations of local people on collecting wild plants gives insights about how local society interacts with nature in general, and more specific with defined parts of it, which is essential for the framing and focusing of management issues [44, 45]. In general, it can be stated that wild plants can have multiple benefits, such as contributing to food security and providing healthy variability in daily nutrition [44, 46], or income from selling, from producing local products, to being a source of pleasure and even adding to cultural identity. European studies [4, 9, 47] reveal two parallel processes regarding the use of wild plants: an abandonment of traditions in the traditional rural agricultural populations, accompanied with a revival of gathering from the wild among educated city-dwellers as the part of a trendy eco-conscious and health-centric lifestyle. In fact, there seems to be a series of motivations, from collecting out of need, through collecting out of habit as a “normal” part of life to a cherished leisure activity. Collecting plants, especially for food, can have negative associations connected to poverty (e.g. “famine food”, war-time food: [8, 24, 38]). Apart from these long-standing negative associations that deter people from gathering, it is also the time consumption of collecting, which often does not fit into modern lifestyle [8, 24, 48]. It might be assumed that the gradient in motivations is correlated to the economic situation of local society, but possibly also to individual financial status [49–51].

In this study, we aim to map and assess the ES of wild plant provision in a rural region in Eastern Europe in central Romania, where plant gathering is still a living tradition and a relevant economic activity. We aim to estimate both for edible plants and medicinal plants collected from the wild (i.e. not cultivated). To map the capacities, we apply a participatory approach relying on the knowledge of the locals as much as possible, exploring if (and how) local, traditional ecological knowledge can still be mobilized in an ecosystem assessment context. This way we also demonstrate that wild plants can be assessed in a way that is fully compatible with the philosophy and recommendations of practical ecosystem service assessments [52–54]. The concrete assessment into which this study was integrated is described by Czúcz et al. [55].

We complement the analysis with a short survey of motivations behind wild food collection of the different layers of local society. Therefore, in the presented work, we assess (i) the capacity of ecosystems within the study area to provide plants for food and medicinal use, (ii) the amount of wild plants collected by locals and their economic value, and (iii) the motivations of the local people for gathering wild food, and we also (iv) analyse the general applicability of ES assessment methods in an ethnobotanical context.

## Methods

### Study area

The study area consists of four partly overlapping Natura 2000 areas (ROSCI0384, ROSCI0297, ROSCI0186, and ROSPA0028) comprising ~ 91,000 ha at the foot of the Eastern Carpathians between 301 and 1080 m a.s.l. in South-East Transylvania, Romania. The main part lies within the county of Mureș while some minor parts extend into the counties of Sibiu and Harghita.

There are altogether ~ 203,000 inhabitants (average population density 68/km^2^), with 13% of the population concentrated in the six cities of the region [56]. A large fraction of the local population belongs to a traditional ethnic minority group speaking Hungarian (called Székely). Accordingly, this is the primary language of the local traditional ecological knowledge for most of the rural population of the region, which we also used during our workshops, surveys and the tables presented (Additional file 1 Table S1). Since the political transition of 1989, the population has been continuously declining due to various reasons (declining birth rates, migration towards bigger cities, emigration). While agriculture is still a dominant source of revenues, official data show that few people earn a living in this sector, subsistence farming is common [50, 51].

South-East Transylvania is a part of Romania in which traditional land use is still very common, while steps towards a modernized agriculture are already taking place [16, 51, 57], GDP is rather low (52% of the EU mean in the study year [58]), unemployment rates are high. In rural Transylvania despite a decline in recent years, many interactions between people and nature are still strongly embedded in the culture and form part of everyday life [59]. As in rural areas in general, here too, the use of provisioning services directly from nature is still a more integral part of life [16, 60, 61].

The natural and semi-natural habitats [62], which are still ample in the region, also represent considerable value from a biodiversity conservation perspective. The vegetation reflects the transitional character between lowland and the mountainous region of the Eastern Carpathians. Forests (37% of the total study site) are usually concentrated at the top of the hills, and are composed of *Quercus robur* L. and *Carpinus betulus* L., which in the higher regions make transitions towards *Fagus sylvatica* L. forests. Pastures (27%) grazed mainly by sheep are scattered with encroached grasslands (regenerating forests in the place of former grasslands) or arable fields. Hay meadows (7%) are dominated by *Agrostis capillaris* L., *Festuca rubra* L., or *Trisetum flavescens* (L.) P. Beauv. In lower sites, there are *Arrhenatherum elatius* (L.) P. Beauv. ex J. Presl & C. Presl. dominated lowland hay meadows and in the vicinity of the Niraj (Nyárád) river traditionally mown lowland hay meadows with *Sanguisorba officinalis* L. are present. Agricultural fields (13%) are typically extensively cultivated, reflecting historical land use and property systems [63]). From the two main rivers, Târnava Mică (Kis-Küküllő) has broad meanders and gallery forests of *Alnus glutinosa* (L.) Gaertn. and alluvial willows.

The most important broad habitat/ecosystem types of the study region were mapped by Czúcz et al. [55]. This map distinguishes 13 ecosystem types (see Table 3) based on land use, land cover, and vegetation characteristics at a spatial resolution of 100 m, and it was used as a key input layer for ES mapping (see, e.g. [53, 55]). For refining the assessment, we also considered further spatial data layers describing various features of the environment, geography, landscape or management (see Table 4). All input data were converted into a 100-m raster of the ecosystem type map. GIS data manipulations were performed in ArcGIS [65], QGIS [66], R [67] with add-on packages *sp* [68], *rgdal* [69], and *raster* [70], and QUICKScan [71], a GIS environment specifically designed to support participatory ES assessment processes.

### Capacity assessment

The study area’s capacity to provide wild plants was assessed in a participatory way relying on the fine resolution map of ecosystem types, and the local traditional knowledge of the residents (rural people, mainly farmers, and local women regularly collecting plants) in three expert workshops (altogether 42 persons in three settlements: Vărgata (Csíkfalva) 15; Eremitu (Nyárádremete) 20; Măgherani (Nyárádmagyarós) 7) in February and March 2016. We mapped the capacity of the region to supply the ES by linking the most important plant species to the ecosystem types. These basic “matrix” models [72] were then complemented with a few adjustment rules reflecting the influence of further relevant environmental factors (e.g. altitude, soil) on the final scores, as described in the following. The two main groups of the target species (wild edible and medicinal plants) were modelled separately according to prior categorizations that were revised after the workshops for final model building. Residents invited to the workshops were selected specifically by recommendations (and with help of the local NGO leading the study) based on their knowledge of wild food, who we thus regarded as local experts in accordance with [73].

**Fig. 1:**
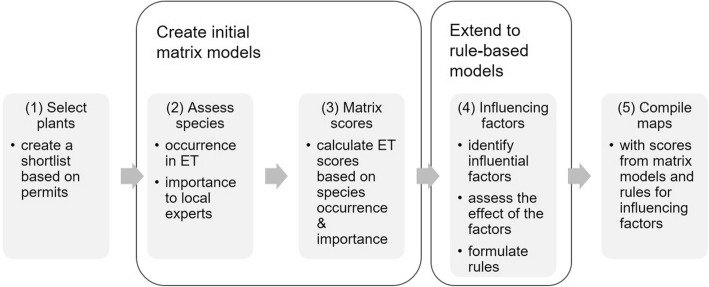
Workflow of modelling the capacity of different ecosystem types to provide wild edible and medicinal plants. ET: ecosystem types

First, we established a list of relevant plant species from the list of the top 15 most collected edible and medicinal plant species in the collection permits issued in 2014 and 2015 by the local Environmental Protection Agencies (APM) of Mureș, Harghita and Sibiu counties (step 1, Fig. 1). For the expert workshops, we created simple leaflets with illustrative photos and the vernacular names of each plant (in Hungarian and Romanian). We also prepared habitat photos for each ecosystem type, and the detailed ecosystem type map of the region to facilitate discussions and to avoid misunderstandings. In the workshops, the selected species were discussed one by one, and the local experts were asked to link the species to the ecosystem types: how frequently/how typically they would find the species there, and score them according to their importance (subjective value)(step 2, Fig. 1). In addition to the species offered, some more species were named by the participants, while some of the presented species were recorded as not collected by any of the three workshop groups. All questions were deliberated until consensus, and only the final scores were recorded.

Information from the workshops was used as shown in Table 1 in order to create two simple matrix models (separately for the edible and the medicinal species). To this end, for each ecosystem type all species that are collected there were weighted with their subjective importance and their frequency of occurrence, species values were summed up for each ecosystem type, and these sums were then rescaled to range between 0 and 10 (step 3, Fig. 1).

**Table 1:** Overview of workshop steps: information gained and further processing for model building (see Fig. 1)

Information from the workshop	Used for	Step
Which species do they know & collect?	> Checking the plant list	2
From which ET within the study area?	> Presence/absence of species within ET	3
Expected occurrence at the habitats (ordinal scale: 1–4, corresponding to the frequency with which the species is expected to occur there)	> Weighting presence/absence for representation of the frequency with which the species occur there	4
The (subjective) value of these species to them (ordinal scale: 1-4)	> Weighting cumulative frequencies within one ET for assessing specific value of ET	4

*ET* ecosystem type

As a last step, the matrix model was extended to a rule-based model [55, 72, 74] (step 4, Fig. 1). The environmental factors to be considered as influencing the provisioning capacity of edible/medicinal plants were identified in a separate expert consultation involving a much smaller number of key experts. Based on the suggestions of the key experts, a small set of “rules” were created in the form of a series of GIS operations that slightly adjust the score values for specific ecosystems. The final ES capacity maps were compiled by applying the rescaled scores and the adjustment rules to the ecosystem type map.

### Actual use valuation

To estimate the economic value of the amount of wild plants actually being collected in the study region as a whole, we multiplied the estimated quantities with estimated market prices for all of the collected species. We started out from the official collecting permits granted by the respective County Environmental Protection Agencies (APM Mureș, Harghita, and Sibiu) per communes and the quantities for the different collected species and plant parts named therein. Plant names and auctors were retrieved from the Integrated Taxonomic Information System (ITIS) (http://www.itis.gov) and the International Plant Names Index (IPNI) (https://www.ipni.org). For our valuation, we assumed that permit quantities reflect actual collection efforts, a method suggested also by Kruse & Petz [19]. In contrast to the capacity assessment, here we used the complete set of species found in the permits so as to make the calculation as comprehensive as possible. As the boundaries of the study area (defined by Natura 2000 areas) do not exactly overlap with the borders of the communes, the total amount of plant material per commune was weighted by the percentage of study area within the communes. The amounts per species and plant parts were then summed up for the whole study area and mean values taken for the years 2014 and 2015.

Prices for the different species and plant parts were collected from regional online vending platforms [75, 76]. Whenever available prices of fresh plant material were used. If only dry weight prices were available, they were multiplied by 0.2 to reflect weight loss during the drying process (derived from the mean value of weight loss factors published by [77–79]). If it was unclear if a price referred to fresh or dry plant materials, we assumed that it was for dry plants: this is the more typical case which also limits the overestimation of the economic value. We found fresh prices for 25 items (species/plant parts) and dry (or ambiguous) prices for 33 items. For the remaining 40 items (which only covered 15% of the total mass of the collected materials) we used a rough imputation method relying on the average of the known prices in three simplified drug categories (H: soft plant parts including *herba*, *folium*, and *flos*; *F*: mature generative parts, including *fructus* and *semen*; and *R*: all woody and underground parts, including *radix*, *rhizom*, and *cortex* according to Ph.Eur [80].).

### Motivations for wild food

In an ES preference assessment survey conducted within the study, local people were asked to prioritize a pre-selected set of 12 ES [55, 81]. In order to study the motivations of local people for collecting wild plants, we also asked them to justify why wild plants from the region were important to them. This allowed us to collect qualitative information on motivations, together with some features of their background (gender, age, profession, education, connection to agriculture). All participants (or their parents in case of children younger than 16) were informed of the study and gave their consent. Altogether, 293 surveys conducted in four different locations within the study area in August 2015 following the methodology described in [81, 82] were analysed. Professions were categorized according to the International Standard Classification of Occupations [83] into nine major groups with four different levels of skills required for work associated with them (“occupational skill levels”, see Additional file 1: Table S2). Connection to agriculture was categorized as “none”, “as a leisure activity”, “subsistence farming“ or as “main income” based on respondents own specifications.

As the answers to motivations were free (non-structured), we categorized them for further analysis by content analysis [84]. We established four categories following the dimensions of well-being (based on [20, 85, 86]): “nutrition/income” meeting the very basic requirements for material welfare (basic physiological needs + financial situation), “healthy” for physical health (free from disease, no exposure to toxins), “pleasure/emotional” relating to mental health, and “habit/tradition” incorporating social relations that may be defined based on certain activities (see Table 5). In case of multiple motivations in a single response, we identified the dominant one, and we coded the response accordingly.

Our intention was to capture possible variations in the attitude towards wild food resulting from differences in socio-economic situation as well as catching the prevalence of tradition-motivated gathering and the emotional motivations lying beneath. Thus, we tested for the influence of the background variables on the ratio of people choosing wild food as an important ES of the region, as well as their influence on the different motivations (chi-squared tests, using R).

## Results

### Capacity

In the workshops, altogether, 37 medicinal and/or edible plant species were scored and linked to ecosystem types by the locals (Table 2). The aggregated importance of each ecosystem type for collecting edible and medicinal plants is shown in Table 3. Broad leaved-forests and wetlands obtained the highest score for providing edible plants, while for medicinal plants orchards were ranked highest, followed by encroached grasslands and pastures. The second best places to collect edible plants were also pastures. On the other hand, forest plantations (Robinia, pine) and intensive agriculture are the worst places to collect wild plants in the region. For orchards, it is mainly *Viscum album* L. and *Crataegus* species that are highly valued as medicinal plants. In broad-leaved forests and wetlands, *Allium ursinum* L., *Corylus avellana* L., *Rubus fruticosus* L., and *Ranunculus ficaria* L. give the habitats’ main value.

**Table 2:** The species of wild edible and medicinal plants recorded as known in the three workshops

Scientific name	Common name (RO)	Common name (HU)	Use (primary; secondary)	Use mentioned	Plant parts collected
*Allium ursinum* L.	Leurdă	medvehagyma, vadfokhagyma	E (M)	Leaves: as stew or soup; cleanses the body, good for the stomach, blood-pressure lowering; after leaves go yellow, the bulb can be dug out and used like onion	herba
(*Acorus calamus* L.)	Obligeană	kálmos			
*Achillea millefolium* L.	Coada șoricelului	cickafark	M	For hip-baths, for women’s complaints, anti-inflammatory	herba
(*Alchemilla vulgaris* L. )	Crețișoară	palástfű			
*Arctium lappa* L.*	Brusture	apró bojtorján	M		herba
*Asarum europaeum* L.*	Pochivnic	kapotnyak, tüdőfű	M	As tea, for losing weight	herba
*Chelidonium majus* L.	Rostopască	vérehulló fecskefű	M	Soothes the eyes, against glaucoma; against warts	herba
*Cornus mas* L.	Corn	som	E	Jam	fructus
*Corylus avellana* L.	Alun	mogyoró	E		fructus
*Crataegus monogyna* Jacq*.*	Păducel	galagonya	M	Good for the heart, elevates blood pressure	fructus
*Equisetum arvense* L.	Coada-calului	zsurló, kannamosó, szuszogó	M		herba
*Hippophae rhamnoides* L.	Cătină	homoktövis, catina	E (M)	Laxative	fructus
*Humulus lupulus* L.	Hamei	komló	E	Fresh shoots as soup (the use of the fruits as medicinal plant was not mentioned)	herba
*Hypericum perforatum* L.	Sunătoare	orbáncfű, jézus burján	M	soothing nerves, for the liver and stomach; "all yellow-flowered are good for the liver"	herba
*Matricaria chamomilla* L.*	Mușețel	kamilla	M	“Rare, used to be more frequent, nowadays not anymore”	flos
Mentha sp.*	Mentă	(vad)menta	M		herba
*Origanum vulgare* L.	Oregano	szurokfű	M	For stomach complaints	herba
*Padus avium* Mill*.*	Cireș pădureț	vadcseresznye, madárcseresznye	E		fructus
*Petasites hybridus* (L.) G. Gaertn., B. Mey. & Scherb.*	Căptălan, busture dulce	acsalapi	M	“Good healing effect, but not very much used”	herba
*Pinus sylvestris* L., *P. nigra* Arnold, *Picea alba* Link	Pin	fenyő (csusza, rügy)	E & M	Against cough, as syrup	herba
*Prunus spinosa* L.	Porumbar	kökény	E	For wine, hard liquor	fructus
*Pyrus pyraster* Borkh.	Păr sălbatic	vadkörte, vackor	E	For hard liquor	fructus
*Ranunculus ficaria* L.	Untișor	salátaboglárka, pipiri saláta, csengő saláta	E	As soup or salad	herba
*Ribes nigrum* L.	Coacăz negru	fekete ribizli, fekete ribiszke	E		fructus
*Rosa canina* L.	Măceș	csipkebogyó, hecserli, seggvakaró	E (M)	Jam, wine, tea; galls against diarrhoea	fructus
*Rubus fruticosus* L.	Mur	szeder	E		fructus
*Rubus idaeus* L.	Zmeur	málna	E		fructus
*Sambucus nigra* L.	Soc negru	bodza	E & M	Flower—syrup; Berries jam; “the same way as black current is used”	flos + fructus
(*Sorbus aucuparia* L. )	Scoruș de munte	madárberkenye, belekenyér, istenkenyere			
*Symphytum officinale* L.	Tătăneasă	fekete nadály	M	Root: heals wounds, very precious; for joint pains, rheumatism	radix
*Tanacetum vulgare* L.	Vetrice	gilisztaűző varádics	M		herba
*Taraxacum officinale* F.H. Wigg	Păpădie	pitypang, gyermekláncfű, cikória, nyúlsaláta	E (M)	(Flowers) as honey; good for the liver	flos + folium
Thymus sp.*	Cimbru	kakukkfű, vadcsombor	M		herba
*Tilia cordata* Mill*.*	Tei	hárs, zádokfa	M	For tea; but “should not be used for the heart”	flos
*Tussilago farfara* L.	Podbal	martilapi, töltike	E	possibly as stew	herba
*Urtica dioica* L.	Urzică	csalán, csikán, csihán	E and M	Purifies blood, as spring cure; strengthens the hair	herba
*Viscum album* L.	Vâsc european	fehér fagyöngy	M	Increases circulation, regulates blood pressure	herba

In paranthesis: species not collected by the workshop participants. Species not presented but added during the workshops are marked with an asterisk. “Use” of species based on information from workshops with “E” for edible and “M” for medicinal use, “primary”: most mentioned use, “secondary”: use of minor importance. Plant parts: the most commonly collected parts of the plants following Ph.Eur [80].

**Table 3:** Ecosystem type categories and their respective scores calculated from workshop scoring

Ecosystem type	Definition	Area %	Assessed productivity for	
			Edible plants	Medicinal plants
Settlement	Villages, outer areas with gardens and single farms	1.8	4	5
Intensive agricultural	Intensive, large arable fields (patches > 10 ha)	0.5	0	3
Extensive agricultural	Mixed agricultural mosaic of small patches of various uses (patches < 10 ha)	12.7	4	7
Pasture	Pastures, grazed grasslands of different degrees of degradation	26.9	8	9
Hay meadow	Hay meadows	6.9	4	8
Encroached grassland	Shrublands, abandoned grasslands encroached with shrubs	7.6	7	9
Wood pasture	Solitary trees in grassland patches	1.7	4	7
Orchard	Abandoned or extensively used fruit tree plantations/vineyards	0.4	5	10
Tree group	Group of trees/small forests/tree rows/galleries along small valleys	3.9	2	5
Pine and spruce forest	Coniferous plantations	1.3	2	0
*Robinia* forest	*Robinia* plantations	0.1	0	0
Broad-leaved Forest	Deciduous forests of native tree species	35.9	10	2
Wetland and water	Major rivers, lakes, and fisheries, including the reed banks	0.3	10	5

**Fig. 2:**
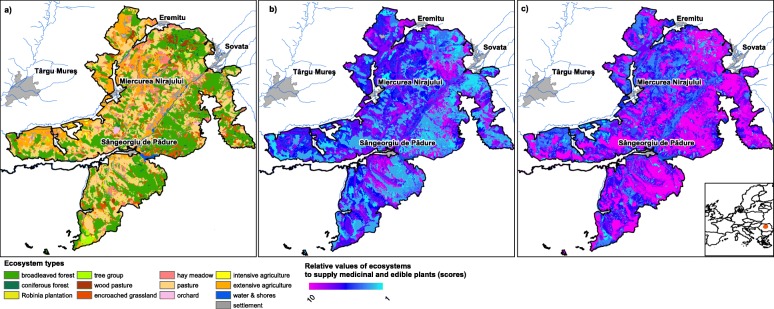
Capacity maps of the study area (**a**) to provide medicinal (**b**) and edible (**c**) plants

In the expert consultations (step 4), soil type, land use in terms of grazing intensity, and generally ecosystem condition were selected as the most influential environmental factors, which should be considered as adjustment rules in the rule-based extension of the matrix model (Table 4). For creating the capacity maps high clay content was counted as positively influencing medicinal herbs, while grazing intensity was considered to be a relevant characteristic of grassland ecosystems (thus only calculated for pastures and wood pastures) and detrimental for the diversity of medicinal and edible plants if too intense. We included also a more general ecosystem condition indicator in our models, which was a biodiversity-based approximation of habitat naturalness. The maps resulting from the models can be seen in Fig. 2.

**Table 4:** Rules applied for modelling and mapping the capacity of ecosystems to provide edible/medicinal plants

Ecosystem service type	Components of the rules					Data sources	
	Ecosystem type	Factor	Direction of effect	Decision space	Effect	Data	Source
Medicinal	All	Soil texture	Clay content less favourable for herbs	Clay content ≥ 30% < 30%	Score <- Score − 1 No change	Soil Map of Romania (SMR-200)	[64]
Edible, medicinal	Pastures, wooded pastures	Grazing intensity	Overgrazing and undergrazing both unfavourable for plant growth and diversity of plants	Grazing > 4 LU/ha 2–4 LU/ha < 2 LU/ha	Score <− Score − 2 Score <− Score − 1 No change	Number of cattle and sheep	Community and municipality administrations
Edible, medicinal	All	Habitat naturalness	The higher general “naturalness” the greater diversity of edible/medicinal plants	Low Medium High	Score <− Score − 1 No change Score <−Score + 1	N2000 species monitoring	[55]

Medicinal: medicinal plants, edible: all edible plants

### Actual use

Collecting permits authorized by the Environmental Protection Agencies of the counties for the years 2014 and 2015 listed 83 plant species altogether (Additional file 1 Table S1). On average, 679 tonnes of plant material was collected per year. The prices for each plant species are listed in Additional file 1 Table S1. Summing up all species, a total economic value of 1.4 million EUR/year (6.4 million RON/year) resulting from harvesting and selling wild food and herbs can be estimated.

### Motivations

**Fig. 3:**
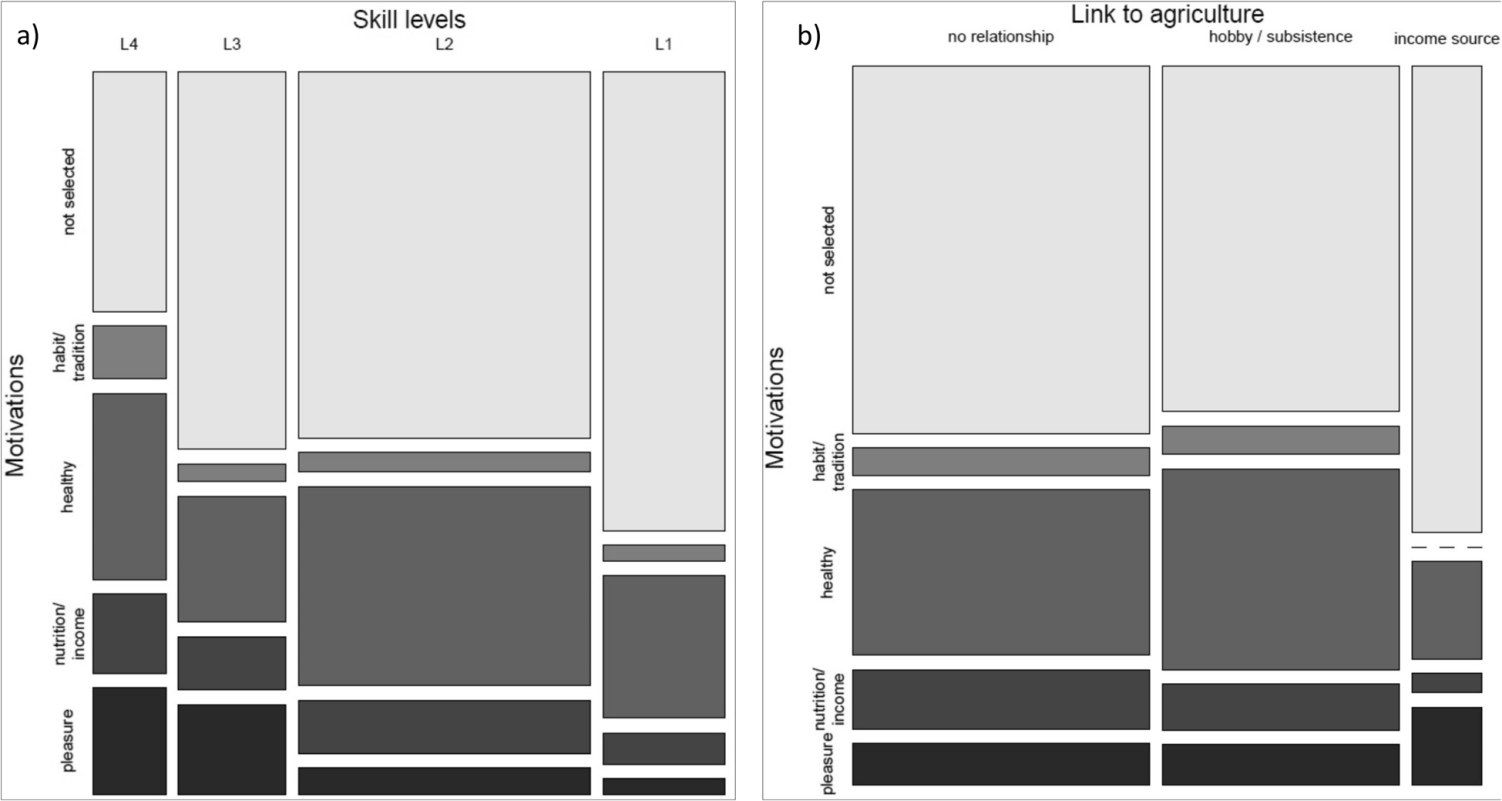
Respondents’ choice to prioritize wild edible or medicinal plants as an important ecosystem service and their justifications. **a** Regarding their occupational skill levels (L1-L4). **b** Regarding their connection to agriculture

There were altogether 132 respondents from the 293 people asked, who gave high importance to the ES of wild edible/medicinal plants (=selected among the first five places at the prioritization exercise). Women selected wild plants at a significantly higher rate than men (chi-squared = 6.748, df = 2, *p* = 0.03425, Table 5). Occupational skill levels were also found to significantly affect the perceived importance of wild plants (chi-squared = 6.3936, df = 3, *p* value = 0.09395): fewer (almost half) of the people from the lowest skill level category considered wild plants as important, while the most educated people rated it important twice as frequently as the middle two skill categories (Fig. 3). None of the other factors examined (age, profession, education, involvement in agriculture, or tourism) seemed to exert significant influence on selecting wild food as important.

**Table 5:** Categories of motivations established based on interviews with their correspondence to dimensions of well-being

Motivation category	Dimension of well-being	Explanation and examples	Percentage of answers (*n*=131)		
			Men (%)	Women (%)	All (%)
Nutrition/income	Material welfare (basic physiological needs + financial situation	Answers mentioning the importance of wild plants for making a living, simple mentionings of just “food”, “you don’t have to pay for it/get it from the shop” or “helps people to survive”	9	8	17
Healthy	Physical health	Answers mentioning physical well-being, wild plants being pesticide-free, "in nature there is medicine" (in general terms, not directly as medicinal use); often in combination with curing, or specifically relating to medicinal herbs	22	38	59
Pleasure/emotional	Mental health	Mentioning direct pleasure or any other kind of emotional bond, or giving subjective reason with “because I like it”; “I like berries” or “I like collecting”	7	8	15
Habit/tradition	Social relations	Answers pointing at the implicitness of collecting, like “because it’s there”, or “medicinal herbs are easy to get”, but also: “base of national identity”, “This is the way, man can survive—living together with Nature”	2	6	8

We also analysed how the respondents had justified their preference for wild plants as an important ES (Table 5). The vast majority (59%) justified their vote with health considerations, which was used for medicinal herbs just as well as for edible plants, seen as non-processed, pesticide-free natural food sources. Women tended to be motivated more by the health aspect than men; however, these differences were not significant. Involvement in agricultural activities has, however, significantly influenced the motivation spectrum of the respondent groups: those who pursued some form of agriculture (either as a leisure activity, or as subsistence farmers), were mainly motivated by the health aspect (38 and 17%, respectively, altogether chi-squared = 34.011, df = 16, *p* = 0.005414, Fig. 3). There were no differences visible in the motivations between people with different age, educational background, or any of the other examined factors.

## Discussion

The collection of wild plants is still very much alive in the eastern parts of Transylvania, at least among the local Hungarian speaking minority, with a widespread recognition of its importance, and large capacities in terms of gathering potential. With our approach of combining official statistical data with local knowledge, we viewed the topic of wild plants as an ES from several different angles resulting in a complex picture that can be represented visually in spatial detail too. The presented method is suitable for capturing local stakeholders’ opinions and incorporate them into the assessment of a provisioning ES of high local relevance. In the following, we also discuss the methodological aspects of our approach from the perspective of generalization towards application in ethnobotany and management.

### Capacity

For most ecosystem types related to traditional land use, several plants (medicinal as well as food-plants) were named by the local experts, whereas for the more intensely used and anthropogenically formed types (settlements, intensive agricultural fields, coniferous, and *Robinia pseudoacacia* L. plantations), it was striking that much fewer plants were named. Lower scores can reflect on both the actually available resources, which are likely to be much less abundant in less natural and more uniform ecosystem types, as well as on the knowledge available to the participants on plants from these ecosystems. Broad-leaved forests support a diverse plant community, which is also utilized and valued by locals. In contrast, *Robinia* plantations are seen to be very homogeneous and lacking any kind of valuable herbs. Pine and spruce plantations were rated also very low as in the investigated lowland-hilly parts they also tend to be relatively free of vegetation, with none of the highly appreciated herbs and berries (e.g. blueberries) of natural coniferous forests characteristic of higher altitudes. Orchards were valued for their secondary herb diversity as well as the mistletoe (*Viscum album*) growing on old fruit trees and used as medicine, while encroached grasslands are populated by bushes of many berry-bearing species *(Rosa canina* L., *Rubus fruticosus*, *Sambucus nigra* L., etc.).

Most earlier ethnobotanical studies on wild food focus on exploring and documenting traditional plant uses and the related ecological knowledge. Such papers follow a primarily descriptive approach, providing lists of plants, discussing traditional or historical plant uses in detail as also stated by Sõukand and Kalle [42]. They draw attention to more recent ethnobotanical works following a more holistic approach including the context in which plant use occurs. Nevertheless, if wild plants are considered as an ecosystem service in an ecosystem assessment context then new challenges emerge. In this case, magnitude and the benefits of collecting should also be quantified. Most studies dealing with wild food in a non-ethnobotanical context consider only on one (or few) key species, which are the most important for the regional economy (e.g. berries in Scandinavian forest: [5, 34], assess habitats and capacities in a qualitative way [61], or focus on one ecosystem, especially on forests [87]).

In this paper we combine these two approaches, introducing taxonomic detail into an expert-based scoring following MAES (Mapping and Assessment of Ecosystems and their Services) recommendation. This can, on one hand, add a quantitative spatial perspective to the local ethnobotanical knowledge and preferences, which on the other hand may lead to more reliable mapping and valuation for ecosystem assessments than assessing/scoring more “general” groups of “wild plants ” as an ES. According to our experiences, decomposing the complex ES of wild food into single species made the scoring easier for the local experts, who were typically locals actively engaged in collecting plants. Our approach involved several scorable elements (the frequency of each species in each ecosystem type, as well as the subjective importance (personal value) of the species), which made them aggregatable at the landscape scale (the species scores aggregated for each ecosystem type, in fact, make up a “matrix model” in an ES assessment terminology, see e.g. [72], or [73]). As a comparison, we also tried the “traditional” MAES approach (asking a few key experts to fill in the “matrix” scores directly (i.e. assign a single aggregated score to the two large thematic plant groups: edible, medicinal) in a series of two test workshops. Nevertheless, according to our experience, the species-based approach seems superior, as it is more efficient and more consistent answers were obtained than if “wild edible and medicinal plants” as such had been evaluated.

Many ES assessments apply a rule-based approach, which is essentially a matrix model augmented with a set of “adjustment rules” which iteratively refine the scores from the matrix model based on further spatial variables (see, e.g. [55, 72]). Adjustment rules can flexibly integrate available environmental data, mobilizing further layers of local expert knowledge at the same time [72, 74, 88]. This is supposed to give better results compared to simply applying a land cover based (matrix) model, which is often considered inaccurate and overly simplistic [89, 90]. Nevertheless, one should be aware of the uncertainties related to the estimates and the results should be regarded as bases from which a dialogue with/between local stakeholders and decision-makers can start [72, 88, 90].

### Actual use

The actual use of an ES is a key element in an ES assessment which constitutes the basis for evaluating the ES’s importance to people, and for the assessment of its economic value, too. Studies on actual use of this ES typically assess individual incomes resulting from picking activities [45, 91]. Harvested quantities can be estimated with surveys and interviews [6, 26, 91, 92], participatory observation [6], or official statistics [33, 34].

In our work we used a particular type of official statistics: collection permits issued by the authorities (as also suggested by Kruse & Petz [19]). This does not include illegal gathering of wild fruits, which is often considered as an important activity in the study area and similar regions of Eastern Europe [26, 59]. In a compilation on South-Eastern European countries’ wild plant collections, Kathe et al. [93] call attention to potential issues with the data sources, which can lead to highly diverging actual use estimations. Permits are issued relatively easily (“as needed” by the retailers for export, according to local informants); thus, for products that get exported, there is little motivation to go undocumented. It is mostly big enterprises, who get the permits and hire private individuals who perform the harvesting. Also, compared with data available for similar regions, it is to be assumed that our estimate is rather above the actual values [93] .

In contrast to earlier calculations [55, 94], here we calculated with data relative to the area of the single communes within the study area (and not just the percentage of all communes taken together within the whole area) and arrived at an estimate of economic value which is almost comparable to that of timber (1.4 million EUR compared to 3.3 million EUR for timber; [18]). But while economic valuation depends much on momentary market prices as well as on calculation schemes, it is of paramount importance to get also a view on the socio-cultural importance in order to get a realistic picture [60, 95, 96].

Wunder et al. [97] suggest using local prices wherever possible, while prices from distant markets might distort actual prices used for valuation, as a number of different level actors are usually involved and transportation costs added. Using prices from online vending platforms as we did, might be placed somewhere in between these two cases, incorporating some of the additional costs, e.g. of transport or advertising). The prices we found were in general higher than the ones mentioned in Albu & Mihalcioiu [98] or compiled as “forest fruits” in the forestry report of the Romanian National Institute of Statistics for the relevant years [56].

### Motivations

There are a number of studies relating differences in wild plant usage depending on a diversity of factors like gender, age or socio-economic status [5, 33, 99], but only few that go beyond that and explore inner motivations of people (e.g. [100]). In our study, almost half of the population considered these gifts that can be picked from nature as important and ranked them among the key services. As in other studies, where women were shown to be often more knowledgeable about collecting and preparing wild edibles, and also more probable to be involved in gathering activities [2, 33, 99], here, they also had a much higher preference for wild food compared to men. These studies found that older people collected more frequently or more species, which we could not confirm in our study. Older people are often seen in closer connection with nature, growing up with collecting as part of everyday life. However, their knowledge is often not well enough transferred to younger generations, who are more used to a modern way of life [101]. People with a higher occupational skill levels, were more likely to have a preference for food and medicine from the wild, which is somewhat in contrast to findings that collecting for subsistence and income are often found in lower income countries as freely obtained nourishment from nature adds to food safety and decreases expenses for food [5, 47, 100]. This preference might be attributed to a selection bias of the interviewees, as individuals of marginalized groups seemed to be less available.

## Conclusion

Combining local ecological knowledge with national statistical data in the context of a regional ecosystem assessment enabled us to give a detailed valuation of the study area’s capacity to provide wild edible and medicinal plants. The combination of ES mapping techniques (matrix workshops/expert scoring), compliant with MAES recommendations, with a more ethnobotanical approach listing plant species, makes it possible to delineate areas (or ecosystem types) of special interest and localize them on a map, which is an important feature for advancing decision-making processes [88, 102]. At the same time, combining the localized knowledge with motivations of different user groups/stakeholders enrichens the basis on which decisions are made, embracing also the aspects of the socially poorest groups, the management of which generally poses the greatest problems, thereby establishing a framework for the sustainable management of the landscape.

## Supplementary information

**Additional file 1: Table S1.** The species, amount and monetary value of plant parts collected in the study area. **Table S2.** Description of the skill levels required for occupations and the ratios of choosing wild food.

## Data Availability

Most of the spatial data used for the modelling are from public data sources (Table 4). The ecosystem type map and the habitat naturalness map of the region, as well as the results of all questionnaires are available from the corresponding author upon reasonable request. All other data are available within the article and its supplementary materials.
